# Linagliptin affects IRS1/Akt signaling and prevents high glucose-induced apoptosis in podocytes

**DOI:** 10.1038/s41598-020-62579-7

**Published:** 2020-04-01

**Authors:** Akira Mima, Toshinori Yasuzawa, Tomomi Nakamura, Shigeru Ueshima

**Affiliations:** 1 Department of Nephrology, Osaka Medical College, Osaka, Japan; 2 Department of Health and Nutrition, Faculty of Health Science, Kio University, Nara, Japan; 3 Department of Food Science and Nutrition, Faculty of Agriculture, Kindai University, Nara, Japan; 4 Department of Applied Biological Chemistry, Graduate School of Agriculture, Kindai University, Nara, Japan; 5 Antiaging Center, Kindai University, Osaka, Japan

**Keywords:** Molecular biology, Nephrology

## Abstract

Diabetes-induced podocyte apoptosis is considered to play a critical role in the pathogenesis of diabetic kidney disease (DKD). We proposed that hyperglycaemia can induce podocyte apoptosis by inhibiting the action of podocyte survival factors, thus inactivating the cellular effects of insulin signalling. In this study, we aimed to determine the effects of linagliptin on high glucose-induced podocyte apoptosis. Linagliptin reduced the increase in DNA fragmentation as well as the increase in TUNEL-positive cells in podocytes induced by high-glucose condition. Furthermore, linagliptin improved insulin-induced phosphorylation of insulin receptor substrate 1 (IRS1) and Akt, which was inhibited in high-glucose conditions. Adenoviral vector-mediated IRS1 overexpression in podocytes partially normalised DNA fragmentation in high-glucose conditions, while downregulation of IRS1 expression using small interfering RNA increased DNA fragmentation even in low-glucose conditions. Because reactive oxygen species inhibit glomerular insulin signalling in diabetes and Kelch-like ECH-associated protein 1 (Keap1)/nuclear factor erythroid 2-related factor 2 (Nrf2) pathway is one of the most important intrinsic antioxidative systems, we evaluated whether linagliptin increased Nrf2 in podocytes. High-glucose condition and linagliptin addition increased Nrf2 levels compared to low-glucose conditions. In summary, linagliptin offers protection against DKD by enhancing IRS1/Akt insulin signalling in podocytes and partially via the Keap1/Nrf2 pathway. Our findings suggest that linagliptin may induce protective effects in patients with DKD, and increasing IRS1 levels could be a potential therapeutic target in DKD.

## Introduction

Diabetic kidney disease (DKD) is the most common cause of chronic kidney disease and end-stage renal disease (ESRD)^1^. Insulin/insulin receptor substrate-1 (IRS1) signalling can increase nitric oxide (NO) production, which is mediated by the PI3K/Akt pathway, thereby increasing anti-inflammatory effects^2^. NO induces vasodilatation and inhibits podocyte apoptosis^3^. Diabetes can inhibit insulin/IRS1 signalling in mesangial and glomerular endothelial cells, probably through the protein kinase C (PKC) β2 pathway^2^.

Vascular endothelial growth factor (VEGF)-A can activate the anti-apoptotic proteins survivin or Bcl-2, thus decreasing endothelial cell apoptosis^4^. Insulin enhances VEGF-A expression, which in turn leads to increased Akt and eNOS phosphorylation, thus protecting against renal cell apoptosis. Furthermore, in our previous study, diabetes could increase podocyte apoptosis in DKD via activation of PKCδ/p38 mitogen-activated protein (MAPK) to enhance Src homology-2 domain containing phosphatase-1 (SHP-1) expression, which in turn resulted in VEGF resistance^5^. Thus, it is likely that the loss of effect of insulin on glomeruli may contribute to the development of DKD.

Incretins are the family of gut hormones including glucagon like peptide-1 (GLP-1) and glucose-dependent insulinotropic polypeptide. Our recent study indicated that a GLP-1 analogue improved diabetes-induced inflammation, preventing mesangial expansion and glomerulosclerosis beyond glycaemic control^6^. As GLP-1 is rapidly degraded by dipeptidyl peptidase-4 (DPP-4), DPP-4 inhibitors that are widely used to treat type 2 diabetes could be a potential treatment for DKD in part because of their pleiotropic actions. A recently published large clinical trial, CArdiovascular safety and Renal Microvascular outcomE study with LINAgliptin in patients with type 2 diabetes at high vascular risk (CARMELINA^®^), established hard renal endpoints using DPP-4 inhibitors for the first time and clearly showed that linagliptin administration prevented the progression of microalbuminuria to overt proteinuria in patients with type 2 diabetes^7^.

Based on the above findings indicating the effect of incretins on insulin signalling, we hypothesised that linagliptin may decrease high glucose-induced podocyte apoptosis through enhancement of insulin/IRS1 signalling in podocytes. Furthermore, it has been reported that interaction between Kelch-like ECH-associated protein 1 (Keap1) and nuclear factor erythroid 2-related factor 2 (Nrf2) can increase anti-inflammatory effects^8^. Previous reports have also reported that diabetes-induced inflammation and oxidative stress could induce intrinsic antioxidant response through the Keap1/Nrf2 pathway^9,10^. Incretin-related drugs decreased inflammatory status and enhanced Nrf2 system in DKD^11^. Therefore, we evaluated whether linagliptin affects this signalling in podocytes.

## Results

### High glucose-induced podocyte apoptosis is suppressed by linagliptin

**Figure 1:**
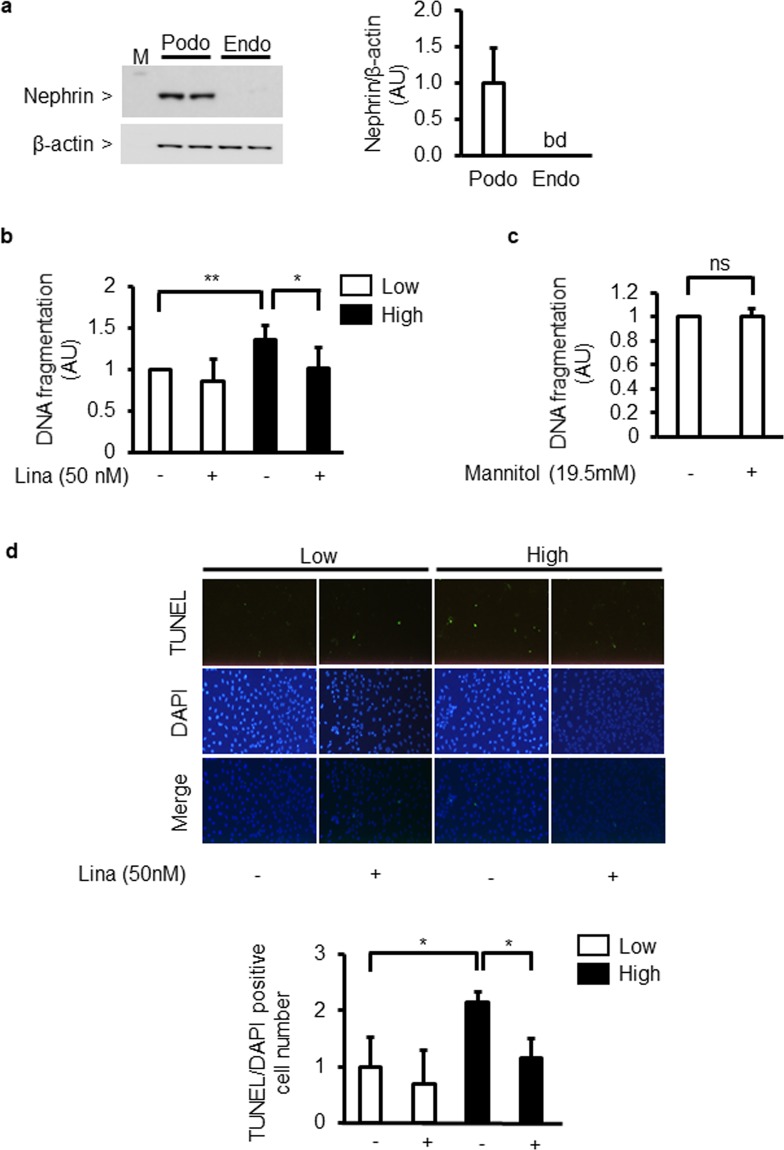
Effect of high glucose and linagliptin on apoptosis in cultured podocytes. (**a**) Immunoblot analyses of nephrin in cultured podocytes and endothelial cells. Podo; podocytes. Endo; endothelial cells. bd; below detection limit. (**b**) DNA fragmentation in podocytes incubated with low glucose (5.5 mM) or high glucose (25 mM) for 96 h in the absence or presence of linagliptin (50 nM). Lina; linagliptin. ^*^P < 0.05. ^**^P < 0.01. (**c**) Effect of D-mannitol-induced hyperosmolality on DNA fragmentation. Low; low glucose. High; high glucose. Lina; linagliptin. ns; not significant. (**d**) Immunocytochemical staining with TdT-mediated dUTP nick end (TUNEL) and 4′,6-diamino-2-phenylindole (DAPI) (merged image). Magnification: X40. Low; low glucose. High; high glucose. Lina; linagliptin. ^*^P < 0.05. These data are expressed as means ± SD. Results are representative of one of three independent experiments.

Nephrin protein expression was recognized in cultured podocytes. In contrast, its expression was not recognized in cultured endothelial cells (Fig. 1a). DNA fragmentation in podocytes exposed to high glucose (25 mM) levels for 96 h increased by 136 ± 17% when compared to that in podocytes exposed to low glucose (5.5 mM) conditions. The addition of linagliptin (50 nM) reduced high glucose-induced podocyte apoptosis by 25 ± 20% (Fig. 1b). However, D-mannitol-induced hyperosmolality did not increase DNA fragmentation (Fig. 1c). Immunocytochemical analyses of podocytes showed that the number of TUNEL/DAPI double-positive cells increased in high-glucose conditions by 216 ± 21% compared to that in low-glucose conditions. However, the addition of linagliptin reduced this increase by 47 ± 13% (Fig. 1d).

### Effect of glucose on DPP-4 expression and linagliptin’s effects on insulin signalling and IRS1

**Figure 2:**
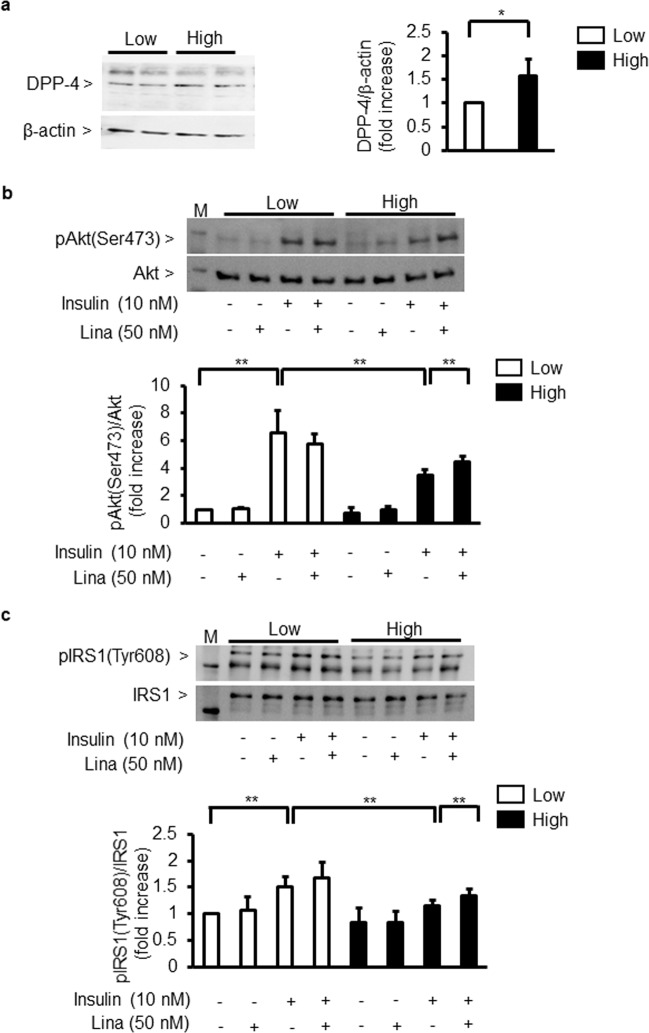
Effect of linagliptin on insulin signalling in cultured podocytes. (**a**) Immunoblot analysis of DPP-4. Podocytes were incubated with low glucose (5.5 mM) or high glucose (25 mM). ^*^P < 0.05. (**b**,**c**) Immunoblot analysis of Akt and IRS1 phosphorylation. After 96 h of exposure to low glucose (5.5 mM) or high glucose (25 mM), podocytes were incubated with insulin (10 nM, 5 min) in the absence or presence of linagliptin (50 nM). (**b**) phospho-Akt. (**c**) phospho-IRS1. Low; low glucose. High; high glucose. Lina; linagliptin. ^**^P < 0.01. These data are expressed as means ± SD. Results are representative of one of three independent experiments.

First, we evaluated whether high glucose could affect DPP-4 expression in cultured podocytes. High glucose (25 mM) levels increased protein DPP-4 expression by 158 ± 36% in podocytes (Fig. 2a).

As Akt activation is known to exert anti-apoptotic effects and it is inhibited in glomerular endothelial cells by diabetes, we characterised the direct effect of glucose levels on insulin signalling and Akt activation in podocytes. Insulin increased phosphorylation of Akt (p-Akt) by 652 ± 189%. However, this increase was reduced by 43 ± 21% in high-glucose (25 mM) conditions. Linagliptin partially normalised insulin-induced p-Akt by 131 ± 20% compared with that in high-glucose conditions without linagliptin (Fig. 2b). Insulin-induced tyrosine phosphorylation of IRS1 (p-IRS1) was increased by 165 ± 38%. In contrast, p-IRS1 was significantly reduced by 29 ± 10% in podocytes incubated in high-glucose conditions compared with that in low-glucose (5.5 mM) conditions. Linagliptin addition reversed the inhibitory effect of high glucose on IRS1 activation by 120 ± 5% (Fig. 2c).

### Effect of linagliptin on insulin signalling in the glomeruli

After 5 week of diabetes, blood glucose levels increased by 389 ± 93% and kidney weight per body weight increased by 150 ± 34% in diabetic SD rats compared to control SD rats (Table 1).

**Table 1:** General characteristics of experimental groups.

	NDM SD	DM SD	DM SD + Lina
Number	5	3	4
Body weight (g)	418.5 ± 31.4	333.3 ± 31.8^**^	289.6 ± 22.6^**^
Blood glucose (mg/dL)	102.2 ± 6.9	398.0 ± 95.3^**^	433.0 ± 82.1^**^
rKW/BW (g/100 g BW)	0.80 ± 0.10	1.20 ± 0.27^*^	1.26 ± 0.20^*^
UAE (µg/mg Cr)	95.6 ± 73.7	695.9 ± 207.8^**^	439.1 ± 60.3^** #^
Serum insulin (ng/mL)	3.32 ± 0.62	0.90 ± 0.16^**^	0.75 ± 0.13^**^

NDM SD, nondiabetic model Sprague-Dawley; DM SD, diabetic model Sprague-Dawley; Lina, linagliptin; rKW/BW, right kidney weight/body weight; UAE, urinary albumin excretion. Data expressed as means ± SD. ^*^P < 0.05, ^**^P < 0.01 vs. NDM SD, ^#^*P* < 0.05 vs. DM SD.

**Figure 3:**
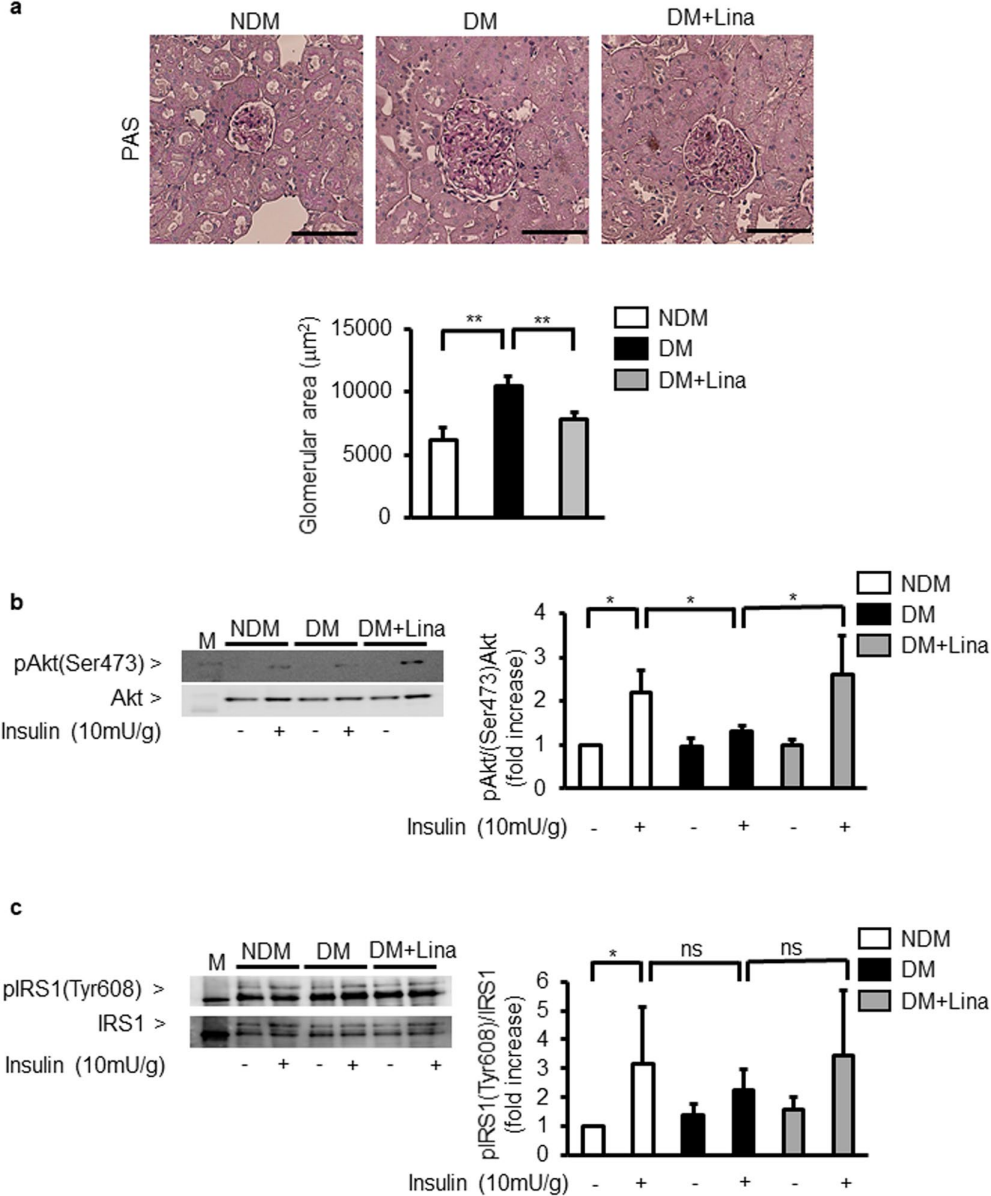
Effect of linagliptin on Akt and IRS1 in the glomeruli of diabetic Sprague-Dawley (SD) rats. (**a**) Representative light microscopic appearance of glomeruli (PAS staining) and morphometric analysis of glomerular area. NDM; nondiabetic SD rats, DM; STZ-induced diabetic SD rats; Lina, linagliptin. Bar=100 μm. ^**^P < 0.01. (**b**,**c**) Representative immunoblots of tyrosine phosphorylation of Akt (**b**) and IRS1 (**c**) from glomeruli. Solubilized glomeruli were subjected to immunoprecipitation followed by immunoblotting. NDM; nondiabetic SD rats, DM; STZ-induced diabetic SD rats; Lina, linagliptin. ^*^P < 0.05. ns; not significant. These data are expressed as means ± SD. Results are representative of one of three independent experiments.

Histological analysis of the glomeruli showed that its area was increased by 171 ± 12% greater in diabetic SD rats than in control SD rats (Fig. 3a).

In the glomeruli, insulin stimulated p-Akt by 219 ± 50% vs control SD rats. In diabetic SD rats, insulin-induced p-Akt levels were reduced by 59 ± 6% compared to control SD rats (Fig. 3b). Moreover, insulin stimulated p-IRS1 by 308 ± 200% vs control SD rats (Fig. 3c). Like p-Akt, insulin-induced p-IRS1 levels were reduced by 71 ± 22% in diabetic SD rats compared to control SD rats. However, no statistically significant differences were found between these groups (Fig. 3c). In addition, we studied linagliptin’s effect on renal function including renal insulin signalling. Linagliptin decreased albuminuria by 64 ± 35% compared to untreated diabetic SD rats (Table 1). Quantitative analysis of periodic acid-Schiff staining showed that linagliptin treatment reduced glomerular area by 27 ± 9% compared to untreated diabetic SD rats (Fig. 3a). In the glomeruli of diabetic SD rats, linagliptin partially normalized insulin-induced phosphorylation of Akt by 201 ± 66% when compared to untreated diabetic SD rats (Fig. 3b). As in the case with p-Akt, linagliptin reversed insulin-induced tyrosine phosphorylation of IRS1 by 154 ± 99% compared to untreated diabetic SD rats. However, there were no statistically significant differences between these two groups (Fig. 3c).

### Effect of IRS1 overexpression on DPP-4 expression and apoptosis in podocytes

**Figure 4:**
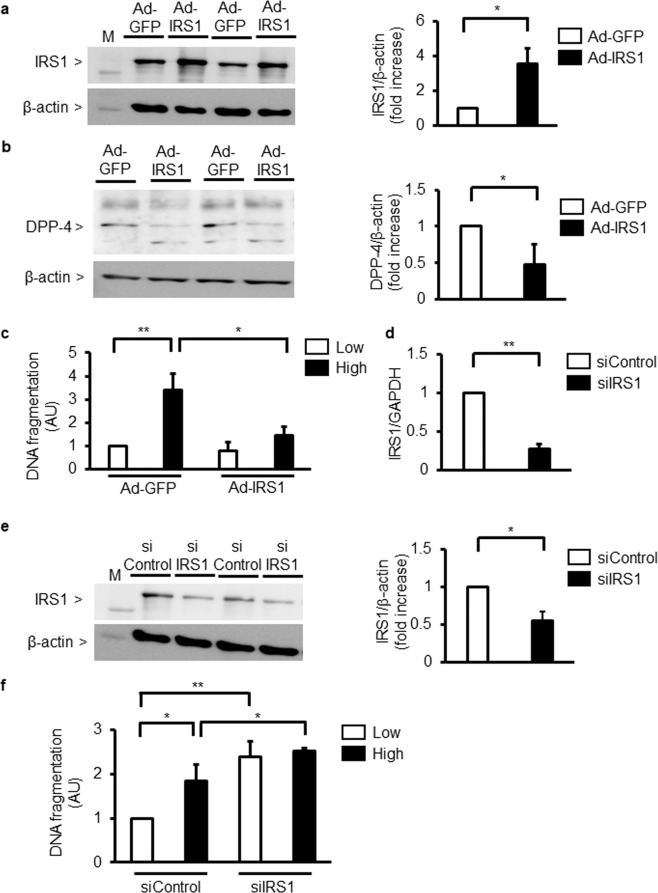
Effect of IRS1 on glucose-induced podocyte apoptosis. (**a**) Immunoblot analysis of IRS1. Podocytes were transfected with adenoviral vectors containing green fluorescent (Ad-GFP or Ad-IRS1) protein. ^*^P < 0.05. (**b**) Immunoblot analysis of DPP-4. Podocytes were transfected with adenoviral vectors containing green fluorescent protein (Ad-GFP or Ad-IRS1). ^*^P < 0.05. (**c**) Podocytes were transfected with adenoviral vectors containing green fluorescent (Ad-GFP or Ad-IRS1) protein and then incubated with low glucose (5.5 mM) or high glucose (25 mM). Podocyte apoptosis was measured by DNA fragmentation. Low; low glucose. High; high glucose. ^*^P < 0.05. ^**^P < 0.01. (**d**) IRS1 mRNA expression in podocytes. Podocytes were transfected scrambled control siRNA (siControl) or small interfering RNA (siRNA) against IRS1 (siIRS1). ^**^P < 0.01. (**e**) Immunoblot analysis of IRS1. Podocytes were transfected with scrambled control siRNA (siControl) or small interfering RNA (siRNA) against IRS1 (siIRS1). ^*^P < 0.05. (**f**) Podocytes were transfected with scrambled control siRNA (siControl) or small interfering RNA (siRNA) against IRS1 (siIRS1) and then incubated with low glucose (5.5 mM) or high glucose (25 mM). Podocyte apoptosis was measured by DNA fragmentation. Low; low glucose. High; high glucose. ^*^P < 0.05. ^**^P < 0.01. These data are expressed as means ± SD. Results are representative of one of three independent experiments.

To determine whether podocyte apoptosis was mediated by IRS1, the expression of IRS1 was increased by Ad-IRS1. Transfection of podocytes with Ad-IRS1increased IRS1 expression by 356 ± 87%, compared to Ad-GFP (Fig. 4a). When podocytes were infected with Ad-IRS1, protein levels of DPP-4 was reduced by 48 ± 28% (Fig. 4b). Infection with Ad-IRS1 significantly decreased high glucose-induced podocyte apoptosis by 54 ± 21% (Fig. 4c). Next, IRS1 expression was downregulated by siIRS1 in podocytes. The addition of siIRS1 in podocytes reduced its expression by 69 ± 3%. Similarly, knockdown of IRS1 with siRNA decreased protein levels of IRS1 by 55 ± 12% compared to control siRNA (Fig. 4d,e). The addition of siIRS1 increased DNA fragmentation by 239 ± 34% even in low-glucose (5.5 mM) conditions (Fig. 4f).

### Effect of linagliptin and the overexpression or knockdown of IRS1 on Keap1/Nrf2 pathway

**Figure 5:**
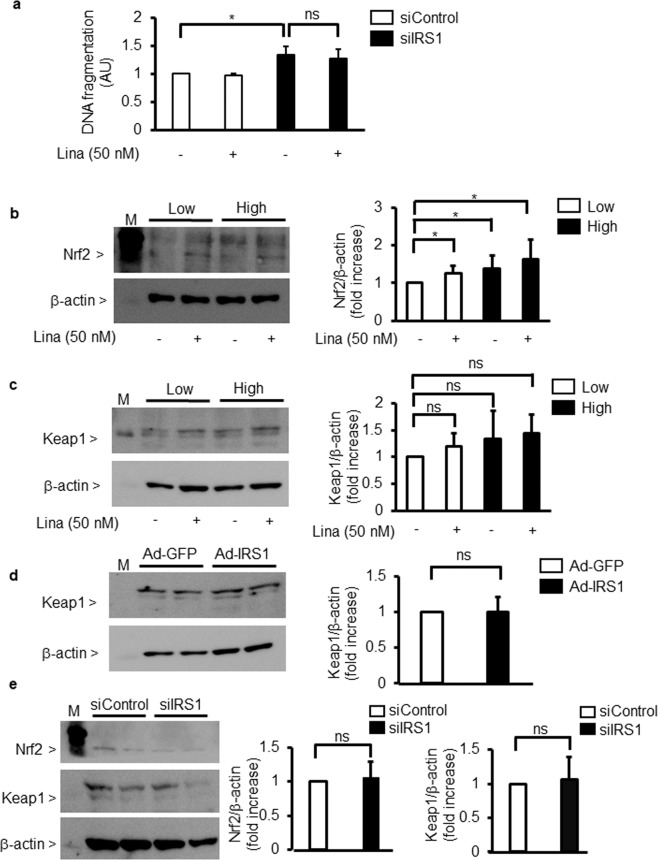
Effect of linagliptin on Nrf2/Keap1. (**a**) Podocytes were transfected with scrambled control siRNA (siControl) or small interfering RNA (siRNA) against IRS1 (siIRS1) in the absence or presence of linagliptin (50 nM). Podocyte apoptosis was measured by DNA fragmentation. Lina, linagliptin. ^*^P < 0.05. ns; not significant. (**b**,**c**) Immunoblot analysis of nuclear factor erythroid 2-related factor 2 (Nrf2) and Kelch-like ECH-associated protein 1 (Keap1). After 96 h of exposure to low glucose (5.5 mM) or high glucose (25 mM), podocytes were incubated with or without linagliptin (50 nM). (**b**) Nrf2. (**c**) Keap1. ^*^P < 0.05. (**d**) Immunoblot analysis of Keap1. Podocytes were transfected with adenoviral vectors containing green fluorescent (Ad-GFP or Ad-IRS1) protein. ns; not significant. (**e**) Immunoblot analysis of Nrf2 and Keap1. Podocytes were transfected with scrambled control siRNA (siControl) or small interfering RNA (siRNA) against IRS1 (siIRS1). ns; not significant. These data are expressed as means ± SD. Results are representative of one of three independent experiments.

The addition of linagliptin (50 nM) did not change DNA fragmentation in IRS1 silencing podocytes (Fig. 5a).

High glucose increased the expression of Nrf2 by 139 ± 35% compared with low glucose. Furthermore, when podocytes were incubated in low-glucose condition, the addition of linagliptin increased the expression of Nrf2 by 120 ± 20% (Fig. 5b). However, the addition of linagliptin or overexpression of IRS1 did not affect protein levels of Keap1 (Fig. 5b,c,d). Lastly, knockdown of IRS1 with siRNA in podocytes did not change protein expression of Keap1 and Nrf2 (Fig. 5e).

## Discussion

In this study, we aimed to investigate the effects of linagliptin on high glucose-induced podocyte apoptosis and found that linagliptin offers protection against DKD through amelioration of IRS1/insulin signalling in podocytes. The possible explanation for linagliptin-induced renal protective effects is that (i) high glucose-induced podocyte apoptosis was recovered by overexpression of IRS1 and (ii) addition of linagliptin reversed high glucose-induced inhibition of insulin-induced tyrosine phosphorylation of IRS1. This is the first study to report the biochemical pathway by which insulin/IRS1 exerts protective action in podocytes.

Podocyte loss is one of the important features of DKD. Podocyte apoptosis is an early stage of DKD progression and therefore, evaluation of podocyte apoptosis could be the best early prognostic marker of DKD^12,13^. Although the mechanism of diabetes-induced podocyte apoptosis is still unclear, previous reports clearly showed that inhibition of insulin and VEGF actions by SHP-1 plays a key role in development of podocyte apoptosis^5,14,15^. In addition, VEGF-induced tyrosine phosphorylation of nephrin was inhibited in the podocytes of diabetic rats^5^.

Similar to glomerular endothelial cells, resistance to insulin signalling and actions for podocytes is also selective for the activation of insulin/IRS1/Akt cascade, which is clearly supported by overexpression of IRS1 using adenoviral vector or knocking down IRS1 using siRNA in the present study. In addition, this study demonstrated that linagliptin reversed high glucose-induced podocyte apoptosis through enhancing insulin/IRS1/Akt signalling.

Previous reports indicated that linagliptin ameliorated renal pathological changes and albuminuria in chronic kidney disease mouse models without affecting blood glucose levels^16^. One possible mechanism of glomerulosclerosis in DKD is endothelial-to-mesenchymal transition (EndMT), which may play a key role in the development of kidney fibrosis. EndMT is defined as a complex process in which cells detach from the endothelial layer resulting in endothelial dysfunction and acquiring a myofibroblastic phenotype^17^. Extracellular matrix, such as type I and IV collagen, or α-smooth muscle actin, which is regulated by transforming growth factor-β (TGF-β) and bone morphogenetic protein 4, is increased in the glomeruli of DKD^18^. Notably, linagliptin decreased the expression of TGF-β-induced EndMT in endothelial cells in diabetic conditions^17^.

It has been reported that both soluble and membrane-anchored forms of DPP-4 are increased in the rodent models of diabetic nephropathy^17,19^. In contrast, another study suggested that the great majority of soluble DPP-4 is the bone marrow rather than kidney^20^. Soluble DPP-4 can activate mannose-6-phosphate/insulin-like growth factor II receptor (M6P/IGF-IIR), increasing the interaction of advanced glycation end product (AGE) with its receptor, RAGE^21^. Membrane-anchored DPP-4 can affect cation-independent mannose 6-phosphate receptor (CIM6PR), activating the transforming growth factor-β (TGF-β)/Smad signalling pathway^22^. Thus, both DPP-4 pathway could develop diabetic nephropathy. Linagliptin may reduce both signalling pathway, decreasing albuminuria and renal fibrosis.

Previous reports indicated that linagliptin could attenuate glomerular area expansion in diabetic rodents without changes in blood glucose, insulin level and body weight^23^. These findings may explain increases in DPP-4 enzyme is recognized in the kidney of diabetes. Further, it is possible that monocyte chemoattractant protein (MCP)-1/CCR2 pathway and invasion of inflammatory cells in kidney can be a pivotal role in developing diabetic nephropathy^24^. Linagliptin could decrease AGE/RAGE, reducing inflammation and reactive oxygen species^25^. Considering these results, linagliptin may have pleiotropic effects beyond glycemic control.

Our study showed that mannitol-induced osmotic stress did not increase podocyte apoptosis. These results mean diabetic conditions, not high osmolarity could increase podocyte apoptosis. Further, our laboratory and others have published data previously regarding the increases in inflammation and oxidative stress, increasing podocyte apoptosis in the kidney of diabetic rodents^5,26^. Clinically, kidney biopsies from patients with type 2 diabetes showed that loss of podocytes^12^. Our previous results indicate loss of insulin signalling in the glomeruli of diabetic rats play a significant role in developing diabetic nephropathy^2^. The novelty of our new findings is due to linagliptin’s effects on insulin signalling, preventing podocyte apoptosis in diabetic nephropathy. The concentration of linagliptin that used in the clinic is up to 100 μg/kg BW, while the concentration we used *in vitro* study was very low. Although convincing, there is a limitation in this point.

Several clinical trials using DPP-4 inhibitors showed decreased rates of microalbuminuria progression. A recent sub-analysis study of Saxagliptin Assessment of Vascular Outcomes Recorded in Patients with Diabetes Mellitus-Thrombolysis in Myocardial Infarction (SAVOR-TIMI) 53 indicated that saxagliptin markedly decreased both overt proteinuria and microalbuminuria^27^. Unlike SAVOR-TIMI 53, the Efficacy, Safety and Modification of Albuminuria in Type 2 Diabetes Subjects Renal Disease with LINAgliptin (MARLINA-T2D) study did not show substantial renal improvements owing to the small sample size^28^. The cardiovascular and kidney clinical trial CARMELINA^®^ clarified the renal outcomes of linagliptin. For the first time, this trial set the renal endpoint using DPP-4 inhibitors: composite renal endpoint [renal death, sustained ESRD, sustained decrease of 40% or more in estimated glomerular filtration rate (GFR)]. The study demonstrated not only cardiovascular safety, but also marked reduction in albuminuria and microvascular composite outcomes, including diabetic retinopathy in patients with type 2 diabetes. However, no significant effects regarding ESRD, death due to kidney disease, and kidney composite outcome were observed in this study^7^.

The glomerular capillary tuft, which is a highly complex and specialised microvascular bed that filters plasma and protects from losing protein to urine, comprises podocytes, endothelial cells, and basement membrane. In contrast, increase in glomerular area could be associated with declining GFR in DKD^29^.

Several large-scale clinical studies, including CARMELINA, reported that DPP-4 inhibitors could ameliorate albuminuria without affecting GFR^7^. These results suggest that DPP-4 inhibitors induced renoprotective effects mainly on podocytes and endothelial cells, rather than mesangial cells. Our present study and previous reports showed that linagliptin directly affected both podocytes and endothelial cells, decreasing albuminuria. However, the effect of linagliptin on mesangial cells, which in turn may affect GFR, is still unclear. Therefore, further study will be needed to clarify this.

Nrf2 is a master modulator of cellular detoxification responses, and the induction of antioxidant responses occurs through the activation of Nrf2 transcription signal^8,30,31^. Bardoxolone methyl, a synthetic oleanane triterpenoid that activates Nrf2, has been found to interact with cysteine residues on Keap1, resulting in Nrf2 translocation to the nucleus. However, a phase 3 clinical trial of bardoxolone methyl in patients with DKD was terminated owing to cardiovascular safety concerns. In contrast, a recent phase 2 clinical trial of bardoxolone methyl (The TSUBAKI study) markedly improved renal function without safety concerns in patients with DKD (American Society of Nephrology Kidney week 2017). Thus, Nrf2 activation seems to be a potential therapeutic target for DKD. Unlike previous reports using DPP-4 inhibitors, our results showed that linagliptin increased protein expression of Nrf2, while Keap1 levels remained unchanged. Linagliptin affected upstream antioxidant signalling, and downregulation of endogenous antioxidant response was observed. Furthermore, linagliptin could not affect protein expression of Keap1, which induces ubiquitination of Nrf2. Previous reports showed that phosphorylation of Akt and GSK3β increased Nrf2 activation without Keap1 system^32^. Thus, linagliptin-induced increase in Nrf2 expression might be mediated by mechanisms other than ubiquitin-proteasome system.

In summary, diabetes inhibited insulin/IRS1/Akt signalling in podocytes, resulting in podocyte apoptosis. Upregulating IRS1 expression or addition of linagliptin reversed this inhibition, thereby protecting against podocyte apoptosis. Thus, linagliptin may induce protective effects in patients with DKD in real-world clinical situations, and increasing IRS1 levels could be a potential therapeutic target in DKD.

## Methods

### Animal studies

All animal protocols were approved by the Kindai University in accordance with the National Institutes of Health guidelines (approval number: KAAG-26-010). We used age-matched male Sprague-Dawley (SD) rats (Shimizu, Kyoto, Japan). Diabetes was induced in 7-week-old SD rats by a single intravenous injection of STZ (50 mg/kg body weight; Sigma, St Louis, MO) in 0.05 mol/l citrate buffer (pH 4.5) or citrate buffer for controls. Blood glucose levels, determined 1 week after the injections by glucose analyser (Sanwa Kagaku, Aichi, Japan) and levels >16.7 mmmol/l, were defined as having diabetes. One week after diabetes, linagliptin (3 mg/kg body weight; Boehringer Ingelheim, Ingelheim, Germany) or vehicle were administered for 4 weeks. Doses of linagliptin was decided based on the previous reports^17,33^. Regular human insulin (10 mU/g; Lilly, Indianapolis, IN) or diluents were injected into the inferior vena cava for 10 min to study insulin signalling.

### Cell culture and reagents

Podocytes from a conditionally immortalised cell line were provided by P. Mundel and cultured as described previously^5^. Briefly, podocytes were cultured in RPMI-1640 (Sigma, St. Lois, MO, USA) medium containing 10% foetal calf serum (FCS), 100 U/mL penicillin, 0.1 mg/mL streptomycin, and 2 mM L-glutamine. For propagation, podocytes were cultivated with a culture medium supplemented with 50 U/mL recombinant mouse γ-interferon (PeproTech, London, UK) at 33 °C with 5% CO_2_. To induce differentiation, the cells were cultured on 10-cm culture dishes coated with type I collagen at 37 °C without γ-interferon. Podocytes were cultured in RPMI-1640 containing 10% FCS. After reaching subconfluence, the cells were exposed to low glucose (5.5 mM + 19.5 mM mannitol) or high glucose (25 mM) with linagliptin (50 nM) in RPMI containing 0.1% FCS for 96 h. Stimulation with insulin (10 nM) was carried out in RPMI containing 0.1% FCS for 5 min. Endothelial cell lines TKD2 were purchased from National Institutes of Biomedical Innovation, Health and Nutrition (Osaka, Japan). Endothelial cells were cultured in RITC80-7 containing 2% FCS. D-(+)-glucose was purchased from Sigma. Linagliptin was provided by Boehringer Ingelheim (Ingelheim, Germany) and doses of linagliptin for i*n vitro* study was decided based on the previous reports^17^.

### Isolation of Glomeruli

Rat glomeruli were isolated from the renal cortex by the sieving method as described previously^2^.

### Adenoviral vector infection

Adenoviral vectors containing green fluorescent protein (GFP, Ad-IRS1) were constructed. These adenoviral vectors were used to infect podocytes as reported previously^2^.

### Small interfering RNA studies

Small interfering RNA (siRNA) against IRS1 (siIRS1) and scrambled control siRNA (siControl) were purchased from Santa Cruz. Differentiated podocytes (0.5×10^5^) were seeded in to 6-cm culture dishes and were grown until they were 60% to 80% confluent. The cells were transfected with siRNA using siRNA transfection system (Santa Cruz) according to the recommended protocol. After 48 h of transfection, cells were exposed to low glucose (5.5 mM) or high glucose (25 mM).

### Immunoblot analysis

Samples were dissolved in 0.5% NP-40, which was used after optimisation studies. Proteins were separated by sodium dodecyl sulphate-polyacrylamide gel electrophoresis (SDS-PAGE). Blots were subsequently incubated with anti-IRS1 (Cell Signaling, Danvers, MA, USA), anti-phospho-IRS (Merck Millipore, MA, USA), anti-Akt (Cell Signaling), anti-phospho-Akt (Cell Signaling), anti-DPP-4 (Abcam, Cambridge, UK), anti-Keap1 (Cell Signaling), anti-Nrf2 (Cell Signaling), and anti-β-actin (Cell Signaling).

### Real-time PCR analysis

IRS1 mRNA was assayed by Real-time PCR and normalized to GAPDH. PCR primers were mouse, IRS1 CACACGGATGATGGCTACATG, GTTTGTCCACAGCTTTCCATAG; mouse, GAPDH ATGTTCCAGTATGACTCCACTCACG, GAAGACACCAGTAGACTCCACGACA.

### DNA fragmentation analysis

DNA fragmentation was measured by quantitation of cytosolic oligonucleosome-bound DNA using ELISA kit, according to the manufacturer’s instructions (Roche Diagnostics, Indianapolis, IN, USA).

### Immunocytochemistry

For immunocytochemical analysis, the cells were fixed with 2% paraformaldehyde and permeabilised with 0.1% Triton X. Apoptotic cells were detected using TdT-mediated dUTP nick end (TUNEL) labelling kit, according to the manufacturer’s instructions (Merck Millipore). 4′,6-Diamino-2-phenylindole (DAPI) staining was performed as described previously^2^.

### Histological studies

Kidney sections for light microscopy analysis were fixed in 4% paraformaldehyde phosphate buffer. Sections were stained with periodic acid-Schiff. Glomeruli were digitally photographed and the images were imported to ImageJ software (National Institutes of Health, Bethesda, MD, USA; https://imagej.nih.gov/ij/) and analysed morphometrically.

### Data analysis

Data are expressed as mean ± standard deviation (SD). Comparisons among more than two groups were performed by one-way ANOVA, followed by post hoc analysis with paired or unpaired *t* test to evaluate statistical significance. All analyses were performed using StatView (SAS Institute, Cary, CA, USA). Values of *P* < 0.05 were considered statistically significant.

## Data Availability

Authors declare that all data is available.
